# The Requirement of WHIRLY1 for Embryogenesis Is Dependent on Genetic Background in Maize

**DOI:** 10.1371/journal.pone.0067369

**Published:** 2013-06-28

**Authors:** Ya-Feng Zhang, Ming-Ming Hou, Bao-Cai Tan

**Affiliations:** Institute of Plant Molecular Biology and Agricultural Biotechnology, State Key Lab of Agrobiotechnology, School of Life Sciences, The Chinese University of Hong Kong, Shatin, N.T., Hong Kong; Ben-Gurion University, Israel

## Abstract

Plastid gene expression is essential to embryogenesis in higher plants, but the underlying mechanism is obscure. Through molecular characterization of an *embryo defective 16* (*emb16*) locus, here we report that the requirement of plastid translation for embryogenesis is dependent on the genetic background in maize (*Zea mays*). The *emb16* mutation arrests embryogenesis at transition stage and allows the endosperm to develop largely normally. Molecular cloning reveals that *Emb16* encodes WHIRLY1 (WHY1), a DNA/RNA binding protein that is required for genome stability and ribosome formation in plastids. Interestingly, the previous *why1* mutant alleles (*why1-1* and *why1-2*) do not affect embryogenesis, only conditions albino seedlings. The *emb16* allele of *why1* mutation is in the W22 genetic background. Crosses between *emb16* and *why1-1* heterozygotes resulted in both defective embryos and albino seedlings in the F1 progeny. Introgression of the *emb16* allele from W22 into A188, B73, Mo17, Oh51a and the *why1-1* genetic backgrounds yielded both defective embryos and albino seedlings. Similar results were obtained with two other *emb* mutants (*emb12* and *emb14*) that are impaired in plastid protein translation process. These results indicate that the requirement of plastid translation for embryogenesis is dependent on genetic backgrounds, implying a mechanism of embryo lethality suppression in maize.

## Introduction

Embryogenesis in flowering plants initiates with fertilization of one sperm cell with the egg, and ends with the formation of a basic plant body including shoot and root meristems. This process requires the functions of many genes. To genetically dissect this complex process, many embryogenic mutants have been isolated, mostly in *Arabidopsis* (*Arabidopsis thaliana*) and maize (*Zea mays*) [1]–[5]. In *Arabidopsis*, such mutants are termed as *embryo defective* (*emb*), which is comprised of mutants with embryo lethality or aberrant seedling [1], [6]. In maize, the embryogenic mutants are classified into *defective kernel*, *empty pericarp*, *small kernel* and *embryo defective* (*emb*) based on the impact on the embryo and endosperm. The *emb* subclass in maize describes seed mutants with specific arrest in embryo development and without significant deleterious impact on endosperm development [5].

A comprehensive analysis of embryo lethal mutants in *Arabidopsis* shows that about 30% EMB proteins have functions in plastids [6]. In particular, mutations in proteins essential for plastid protein translation cause embryogenesis arrest, *e.g.* plastid ribosomal proteins (PRPs) and several pentatricopeptide repeat proteins (PPRs) [6]–[8]. In tomato (*Solanum lycopersicum*), the *defective chloroplast and leaf* (*dcl*) mutant that is defective in the processing of plastid 4.5S rRNA, shows embryogenesis arrest at the globular stage [9]. In maize, mutations in plastid *RPS9* (*Lem1*) and *RPL35a* (*Emb8516*) cause early embryo abortion [10], [11]. Recently we analyzed *Emb12* and *Emb14* in maize which respectively encode the translation initiation factor 3 and an YqeH homolog in plastids, both of which are required for the formation of translation machinery ([12], Li C. and Tan, B.C., unpublished data). Mutations in either genes cause embryo development arrest at transition stage. These results demonstrate that plastid protein translation is essential to embryogenesis.

However, there is also evidence indicating that plastid translation is not equally important to embryogenesis between *Arabidopsis* and maize. For example, the nucleus encoded CRS2-associated factor 2 (CAF2) is required for group II intron splicing in *rps12* in chloroplasts [13], [14]. Loss of function in CAF2 causes plastid ribosome deficiency in both maize and *Arabidopsis*, but in *Arabidopsis* it results in embryo lethality whereas in maize it allows normal embryogenesis and conditions an *albino* seedling phenotype. Similar results were reported with plastid PPR2, PPR4, PPR5 and THA8 (thylakoid assembly 8) proteins [8], [15]–[19]. All of these proteins function in plastid RNA metabolism, however, null mutations in these genes cause plastid ribosome deficiency. Again, mutations of the orthologs in *Arabidopsis* cause embryo arrest and lethality, whereas in maize the loss of their functions allows normal embryogenesis and conditions albino seedlings.

Plastids are the sites of many important processes such as photosynthesis, and biosynthesis of fatty acids, amino acids and several phytohormones, *etc*. Impairment in plastid protein translation may impact these processes, causing embryo developmental arrest. For example in *Arabidopsis*, the plastid genome contains *accD* gene coding for the β-carboxyl transferase subunit of the plastid heteromeric acetyl-CoA carboxylase (ACCase) [20]. Heteromeric ACCase produces malonyl-CoA that is used for de novo biosynthesis of fatty acid in plastids [21]. Lipid synthesis in plastids is proved to be essential for embryo development [22]–[24]. Bryant *et al.* (2011) proposed that the expression of *accD* gene in plastids is the requirement of plastid translation for embryogenesis in *Arabidopsis* [6].

The nuclear encoded WHIRLY1 (WHY1) proteins are known to have versatile roles. In barley (*Hordeum vulgare*) and *Arabidopsis*, WHY1 is dual-localized in both the nucleus and the chloroplast [25]–[27]. In the nucleus, it was shown to act as a transcription activator for pathogen related gene expression in *Arabidopsis* and potato (*Solanum tuberosum*) [25], [28], and repressor for the kinesin-like protein 1 (KP1) in *Arabidopsis* [29]. It was also implicated in modulating the homeostasis of telomere length in *Arabidopsis* [30]. In chloroplasts, WHY1 is implicated in the repair of plastid genome, and is necessary for the genome stability in maize and *Arabidopsis* [31], [32]. In maize, WHY1 is also essential for the biogenesis of plastid ribosome. The severe loss of function allele *why1-1* in the standard genetic background and B73 conditions albino seedlings [33], [34].

Here we report the characterization of an *embryo defective 16* (*emb16*) mutant in maize. The *emb16* mutant was isolated from the UniformMu population in near isogenic W22 genetic background [4]. The mutation causes embryo development arrest at transition stage. Molecular cloning indicates that the *emb* phenotype is caused by a null mutation of the *Why1* gene. Further genetic analyses demonstrate that the requirement of WHY1 function for embryogenesis is dependent on the genetic background. And this dependence exists in two other embryo defective genes that affect plastid translation. These results indicate that the requirement of plastid translation for embryogenesis may not be related with the expression of maize plastid genome, and it should be independent of the fatty acid synthesis pathway [6].

## Materials and Methods

### Plant Materials

The mutants of *emb16*, *emb12-1*, and *emb14-1* were derived from the UniformMu transposon tagging population which is created by introgressing the Mutator (*Mu*) active line into a W22 inbred background [4]. All these mutations were maintained in a W22 genetic background. For developmental analyses and population generation, the plants were grown in the Gene Garden of the Chinese University of Hong Kong and manually pollinated. The *iojap* (*ij*) seed stock and the inbred lines were provided by the Maize Genetics Cooperative Stock Center. The seeds of heterozygous *why1-1* and *why1-2* were kindly provided by Dr. Alice Barkan (Oregon State University).

### Histological Analysis of the *emb16* Seed Development

Developing WT and mutant kernels were harvested from the self-pollinated segregating ear at 5, 6, 7, 10, 14, 21, and 27 days after pollination (DAP). The kernels were cut along longitudinally into three equal parts. The central slice containing the embryo was fixed in 4% (w/v) paraformaldehyde for overnight at 4°C [35]. The fixed material was dehydrated in a graded ethanol series, infiltrated and embedded in paraffin, sectioned at 8 to 10 µm with microtome (Jung Biocut 2035, Leica, Germany), and mounted on slides as described [35]. The sections were then deparaffinized, stained with safranin O and fast green [36], and observed with microscope (Eclipse E80i, Nikon, Japan).

### Transmission Electron Microscopy Analysis

The *emb16* and WT embryos were dissected from a segregating ear at 6, 7, 8, and 14 DAP using stereomicroscope. At 6 DAP, the *emb16* embryo was distinguished from the WT by the size and structure of the embryo proper, and confirmed by PCR genotyping on the endosperm tissue. For chloroplast structurally analysis, sections 1 cm below the tip of the second leaf from two-leaf stage seedlings were collected and cut into small pieces. The fixation, dehydration, infiltration, and embedding of embryo and leaf tissues were performed as described [37]. Ultrathin sections of 70 nm were cut using diamond knife (Diatome, Electron Microscopy Sciences, USA) and ultramicrotome (Ultracut S, Leica, Germany), and lifted onto 2 mm copper grids. Grids were stained in uranyl acetate and lead citrate prior to observe with transmission electron microscope (H-7650, Hitachi, Japan).

### Southern Blot Analysis and Cloning of *Mu* Flanking Sequence by TAIL-PCR

Maize genomic DNA was extracted from two-leaf stage seedlings using the urea extraction method [38]. About 10 µg genomic DNA was digested with appropriate restriction enzymes and resolved on 0.7% (w/v) agarose gel. DNA fragments were transferred to nylon membranes (Amersham, UK) and hybridized with probes as previously described [39]. The probe was labeled by [α-^32^P]dCTP (3,000 Ci mol^−3^) using the DNA labeling beads (Ready-To-Go, GE Healthcare, USA). The *Mu1*/*Mu2* probe was derived from the *HinfI* fragment of the *Mu1* element, which contains the internal sequence without the terminal inverted repeat (TIR) region.

The improved high efficiency TAIL-PCR method [40] was adapted for amplification of the *Mu1*/*Mu2* flanking sequence in the 7.5 kb *EcoRI* fragment that was identified in the co-segregation analysis. *EcoRI* digested genomic DNA (*emb16*/+) was resolved on 0.7% (w/v) agarose gel. DNA fragments with size from 7.3 to 7.7 kb were recovered and used as PCR template. *Mu1* and *Mu2* specific nested primers were designed based on the internal sequences of the *Mu1* and *Mu2*, with Mu2-F1 and Mu2-F2 for the forward direction and Mu2-R1 and Mu2-R2 for the opposite direction (Table S1). Four arbitrary degenerated primers (AD3-1 to AD3-4) were designed partially based on the report by Liu and Chen (2007). The 5′ embedded primer in these four AD primers (i.e. AD3 primer) was derived from a non-maize sequence (Table S1) and a BLAST analysis against the maize genomic sequence in the GenBank did not identify any priming site. Three rounds of TAIL-PCR were carried out. In the first round, *Mu2* primers in combination with AD3-1, AD3-2, AD3-3, or AD3-4 were used. The second and third rounds used the nested *Mu2* or TIR primers (TIR6 or TIR8) in combination with AD3 (Table S1). The TIR8 primer is a mixture of the TIR8.1, TIR8.2, TIR8.3 and TIR8.4 primers in a 2∶4:4∶1 ratio [41]. The PCR conditions were according the report, with minor adjustment of temperatures depending on the primers and DNA polymerase. In most cases, several fragments were amplified after the second or third round of TAIL-PCR. These fragments were recovered and ligated into vector (pCR2.1-TOPO, Invitrogen, USA), and sequenced.

### Fractionation of Chloroplasts and Nuclei from Maize Leaves

The fractionation of chloroplasts was based on the procedure as previously reported with modifications [42]. About 25 g leaves of two-leaf stage W22 seedlings were chopped and ground in a blender in ice cold 250 ml grinding buffer [GR buffer; 50 mM HEPES-KOH pH 7.5, 330 mM sorbitol, 1 mM MgCl_2_, 1 mM MnCl_2_, 2 mM EDTA, 5 mM Na-ascorbate and 1% (w/v) bovine serum albumin]. The grinding was conducted in four 10 second pulses at low speed setting. The ground was filtered through one layer of pre-wet Miracloth (Calbiochem, USA). The solution was centrifuged at 3,000 g for 8 min at 4°C. The pellet, which contains chloroplasts, was resuspended in 5 ml ice-cold GR buffer and laid on top of a prepared Percoll gradient (50% 2× GR buffer and 50% Percoll), and centrifuged at 6,500 g for 15 min. The top band was discarded. The lower band was collected and diluted 3 folds with import buffer (50 mM HEPES-KOH pH 8.0, 330 mM sorbitol). The chloroplasts were centrifuged down at 2,600 g for 8 min, then washed by resuspending in 15 ml import buffer and centrifuged at 2,600 g for 4 min. The chloroplasts were suspended in an appropriate volume of import buffer to get about 1 mg chlorophyll per ml.

The isolation of intact nuclei from maize leaves was conducted as previously reported [43]. The integrity and purity of the nuclei were checked by microscopic observation.

### RNA Extraction and RT-PCR Analysis

For RNA isolation, the seedlings homozygous for *why1-1*, *why1-2*, or *why1-3 *were genotyped by PCR using primers of Emb16-R2 in combination with Mu2-F1 for *why1-3*, and TIR8 for *why1-1* and *why1-2* (Table S1). Leaf RNA was extracted from the middle of the second leaf with RNeasy mini kit (Qiagen, Germany). For RT-PCR, the first-strand cDNA was synthesized using the PrimeScript® reverse transcriptase kit (Takara, Japan). Emb16-R1 in combination with Emb16-F2 or Emb16-F1, which cross the *Mu* insertion site were used to amplify the *why1* transcripts in *why1-1* and *why1-3* albino leaves (Table S1).

### Protein Extraction and Western Blotting Analysis

Proteins from maize leaves of WT, *why1-1*, *why1-2* and *why1-3 *were prepared as previously described [44], and the concentration was quantified by protein assay kit (Bio-Rad, USA). Ten microgram protein of each sample was subjected to Western blot as described [45].

## Results

### Embryo Development Arrest in the *emb16* Mutant

**Figure 1:**
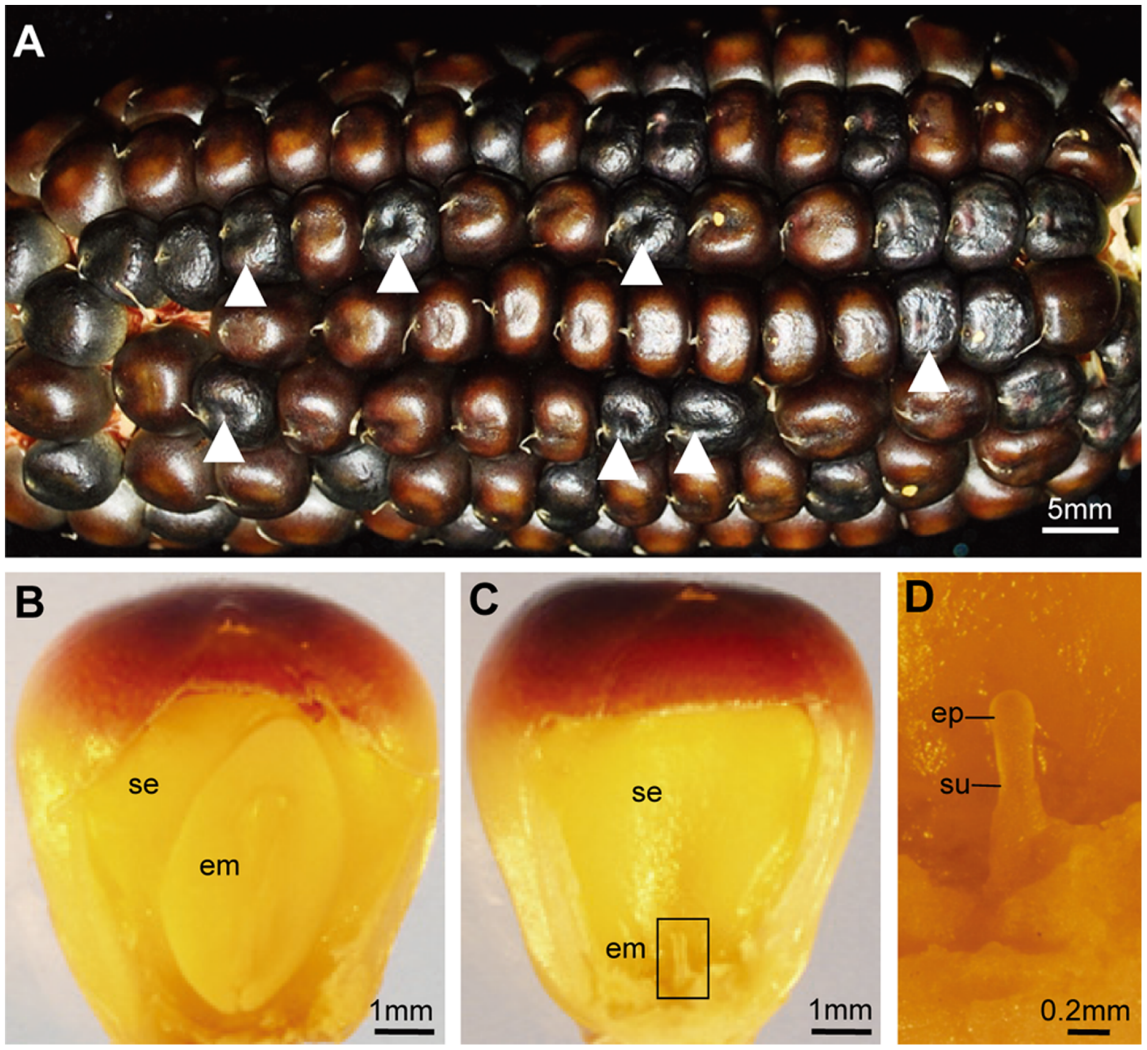
Phenotype of *emb16* mutant in W22 inbred background. (A) An ear segregating the *emb16* mutant. Arrows point to the *emb16* mutant kernels. (B) Mature WT kernel. (C) Mature *emb16* mutant kernel. (D) Enlarged view of the mutant embryo in (C). se: starchy endosperm; em: embryo; ep: embryo proper; su: suspensor. Scale bars as indicated.

The *emb16* mutant is monogenetic, recessive (288∶91, wildtype:*emb*, 3∶1, p>0.50) and homozygous lethal. The mutant kernel is about the wildtype (WT) size and often with dark pigmentation, a feature that is observed in kernels of many maize *emb* mutants (Figure 1). The endosperm appears to be normal and is filled with starch. However, the embryo is defective. At maturity, the mutant embryo is arrested at transition stage, only develops a pre-embryo proper and a suspensor [46]. The WT at this stage develops an embryo with primary shoot, root and scutellum. We attempted to rescue the embryo by culturing it in the MS medium at 7, 10, 14, and 20 DAP, but all attempts were not successful.

**Figure 2:**
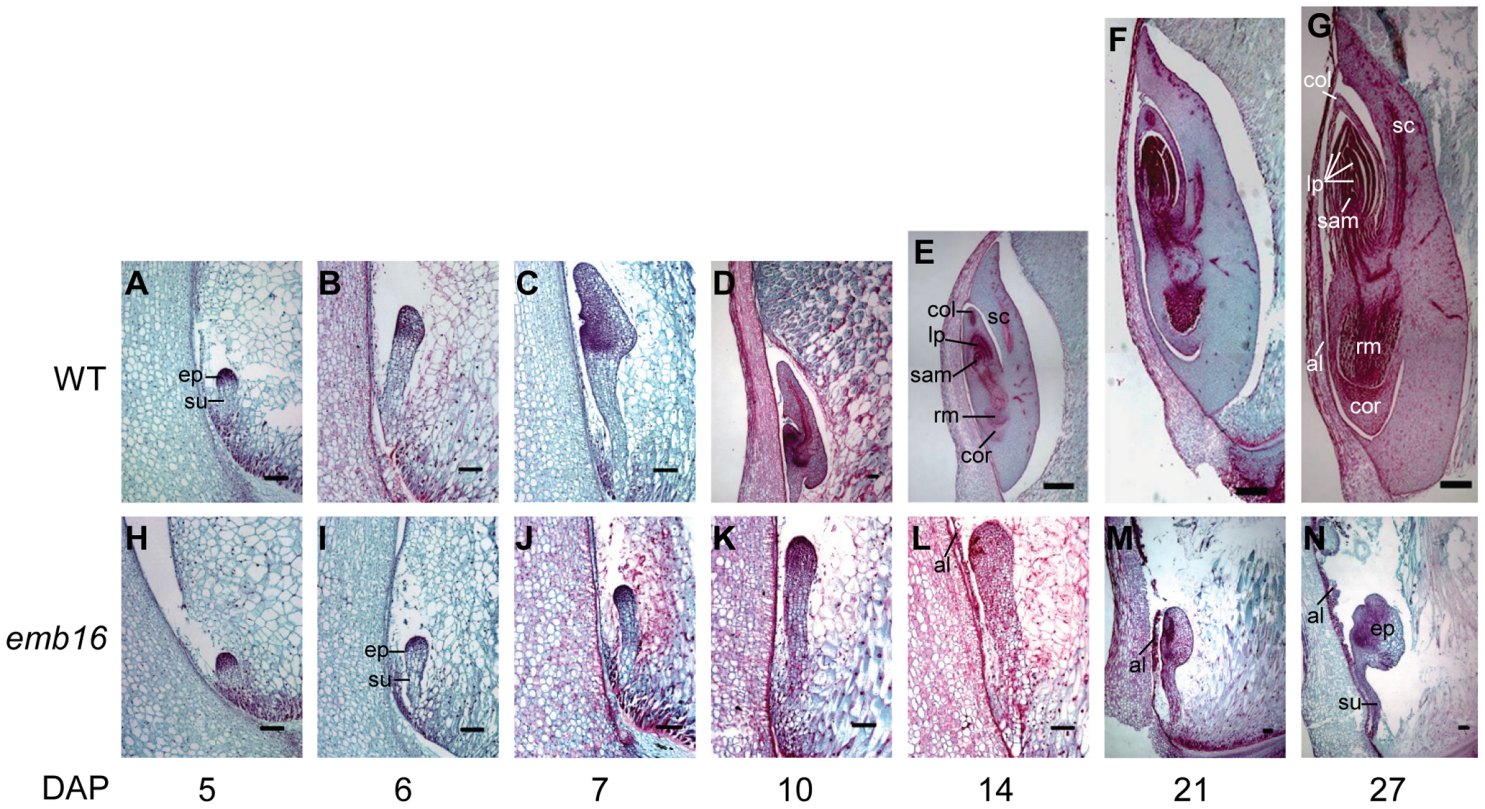
Embryo development in *emb16* mutant. The WT and *emb16* mutant kernels from segregating ears were sectioned from 5 to 27 DAP. Longitudinal sections of maize kernels were stained with Safranin O and fast green. At 5 and 6 DAP, the *emb16* embryos were distinguished from the WT by the size and structure of embryo proper. al: aleurone cell; col: coleoptile; cor: coleorhiza; ep: embryo proper; lp: leaf primordia; rm: root meristem; sam: shoot apical meristem; sc: scutellum; su: suspensor. Scale bars: E, F, and G = 1 mm; others = 0.1 mm.

To determine the impact of *emb16* mutation on embryogenesis, we compared the embryo development process between the *emb16* mutant and the WT. For a precise comparison, we analyzed the mutant and its WT siblings in a segregating ear since these seeds developed at identical conditions. The mutant embryos were identifiable from the WT at as early as 5 DAP by the size and appearance (Figure 2, A and H). Under our growth condition, the WT embryo establishes apical-basal axis at 5 DAP and forms primary scutellum, coleoptile and shoot and root primordia at 10 DAP (Figure 2, A–D). By 14 DAP, the embryo develops all the structures of a mature embryo, but at about half the size (Figure 2E). By 21 DAP, the embryo has developed primary root and 3 to 4 primary leaves (Figure 2F). By 27 DAP, the embryo enlarges and forms 4 to 6 primary leaves (Figure 2G). In contrast, the *emb16* embryo establishes the apical-basal axis at 6 DAP, and appears to differentiate until 10 DAP with the increased cell density at the adgerminal face of the embryo proper (Figure 2, I–K). At 14 DAP, the mutant embryo ceases differentiation and shows signs of degeneration (Figure 2L). At 21 and 27 DAP, the embryo forms a tumor like head structure which is still attached to a suspensor (Figure 2, M and N). This developmental analysis indicates that the *emb16* mutation arrests the embryo development at the transition stage as previously defined by Abbe and Stein (1954).

Although the *emb16* endosperm appears normal, the aleurone cells are not. At 14 DAP, the aleurone cells in the adgerminal face of the *emb16* kernel divides inwards (Figure 2L; Figure S1D). As the kernel develops, the aleurone cells continue the inward division, causing disorganization of aleurone cell layer (Figure 1, M and N; Figure S1, E and F). From this observation, we conclude that the *emb16* mutation also affects the endosperm cell development, but only at a lesser extent. This conclusion is consistent with the dark pigmentation in the mutant kernel which is likely from the aleurone cells.

### Ultrastructure of the *emb16* Embryo Cells

**Figure 3:**
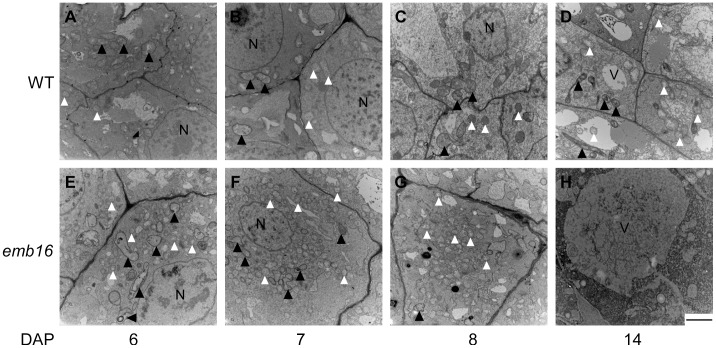
Embryo cell development in *emb16* mutant. The WT and *emb16* mutant kernels from segregating ears were sectioned from 6 to 14 DAP. At 6 DAP, the *emb16* embryo was distinguished from the WT by the size and structure of embryo proper using stereomicroscopy and confirmed by the endosperm genotyping. The ultrastructural observation of embryo cells in the *emb16* mutant is from the embryo proper cells and in the WT is from the embryo proper cells (6 DAP) or shoot meristem cells (7–14 DAP). The content of the embryo proper cells is different from that of suspensor cells, which contain more starch granules and vacuoles. Similar cell contents were observed in cells of WT shoot meristem, leaf primordia, and coleoptile. Empty arrow heads point to mitochondria, and filled arrow heads point to proplastids. N: nucleus, V: vacuole. Scale bars = 2 µm.

To further characterize the effect of the mutation on embryo cell development, we analyzed the ultrastructure of the *emb16* embryo during the early stages of seed development by Transmission Electron Microscopy (TEM). Proplastids in the WT embryo at 6 DAP contain almost no inner membrane structure, and this feature is quite common to proplastids in the embryo proper cells (Figure 3A; Figure S2A). In the *emb16* mutant, the inner structure of proplastids is varied, some with thylakoids and/or vesicle-like structures, others with starch granules. And the number of mitochondria increased compared with the WT (Figure 3E; Figure S2D). From 7 to 14 DAP, no significant change in the number of mitochondria was observed in the cells from shoot meristem as the WT embryo differentiated and the inner structure in proplastids developed with increased membrane system (Figure 3, B–D; Figure S2, B and C). At 14 DAP, the formation of linear thylakoid is observed in the WT (Figure 3D). In the *emb16*, an increased number of mitochondria is still observed at 7 and 8 DAP (Figure 3, F and G; Figure S2, E and F). At 14 DAP, the embryo cells become vacuolated (Figure 3H), a sign of cell death [11], [47]. These observations suggest that the *emb16* mutation causes abnormal formation of the inner structure of proplastids, and intriguingly an increased number of mitochondria. The mutant embryo cells undergo cell death at 14 DAP.

### Cloning of *Emb16*

**Figure 4:**
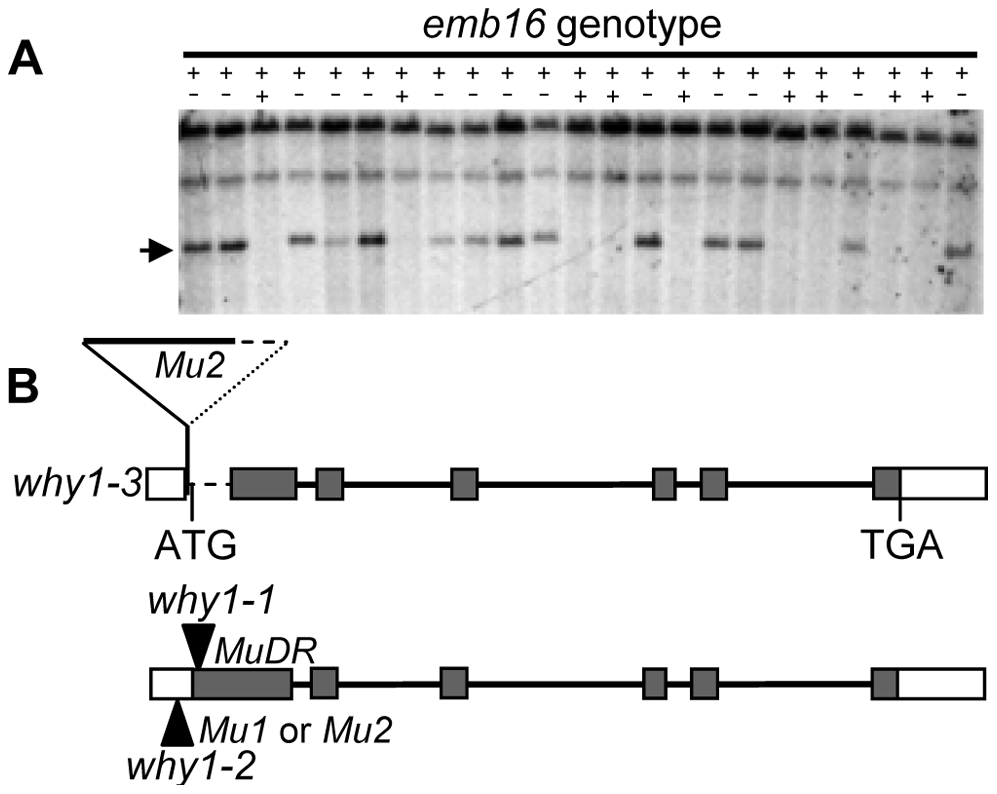
Cloning of *Emb16* gene. (A) Southern analysis of individual plants from an *emb16* segregating family by using a *Mu1*/*Mu2* specific probe. “++”, WT and “+−”, heterozygote for the *emb16* mutation. The genomic DNAs were digested with *EcoRI*. Arrow points to a 7.5 kb fragment that co-segregates with the *emb16* mutation. (B) Gene structure of *Why1* gene and *Mu2* insertion site in the *why1-3* allele. A fragment covering 380 bps *Mu2* and 245 bps *Why1* (dotted line) was deleted in the *why1-3* allele. Exons are boxes and introns are lines. Translated regions are filled boxes. Triangles are *Mu* insertions in the *why1-1* and *why1-2* allele [33]. ATG: translation start codon, TGA: translation stop codon.

The *emb16* mutant was isolated from the UniformMu population, which was created by introgressing the *Mu* active line into the W22 inbred background [4]. The active *Mu* line was from Donald Robertson’s collection and had been backcrossed to W22 for six times when the *emb16* mutation was isolated. After the isolation, the mutant was backcrossed two times to W22 again in an effort to reduce the active *Mu* copy number. As a result, the *emb16* mutation used in this study is in nearly isogenic W22 background (99.6%). To identify the mutation, co-segregation analysis was used. DNA hybridization by using a *Mu1*/*Mu2* specific probe identified a 7.5 kb *EcoRI* fragment that co-segregated with the mutant phenotype (Figure 4A). Increasing the population to 60 individuals showed the same linkage, suggesting that this *Mu* insertion is either the cause of or tightly linked to the *emb16* mutation. To reveal the *Mu* flanking sequence, we improved the previous MuTAIL-PCR [41] in three aspects. First, we adapted it with an improved TAIL-PCR protocol [40] which increases the efficiency while reduces the reaction number to three. Second, we used size selected genomic DNA enriching the 7.5 kb *EcoRI* fragment. Third, we employed *Mu1*/*Mu2* specific primers coupled with degenerate primers (referring to the Materials and Methods). The improved protocol was proven robust in extracting *Mu* flanking sequences, saving efforts in comparison to screening a genomic library. Sequencing results indicate that a *Mu2* element is inserted in the *Why1* gene (acc: EU595664) which was previously identified [33]. *Why1* contains six exons and the *Mu2* element is inserted in the first exon (Figure 4B). The insertion also caused a deletion of 380 bps in the *Mu2* element and 245 bps in the *Why1* first exon. The deletion includes the translation start codon of the *Why1* gene, suggesting that this allele may be null.

**Figure 5:**
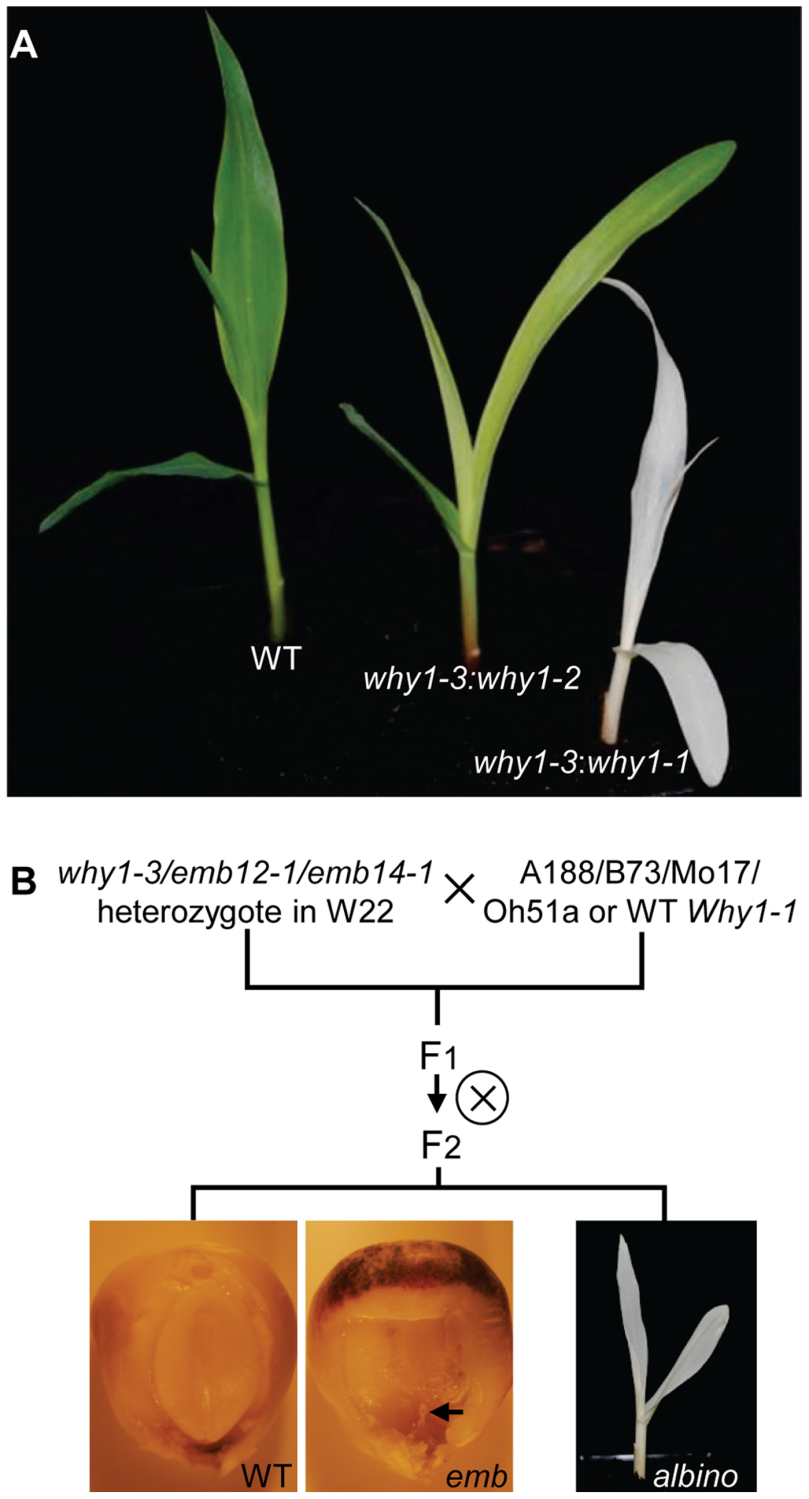
Phenotypic expression of *why1-3*, *emb12-1*, and *emb14-1*. (A) *albino* seedlings from the crosses between heterozygous *why1-3* and *why1-1 or why1-2*. (B) The *emb* kernel and *albino* seedling from the F2 progenies of the crosses between *why1-3*, *emb12-1*, or *emb14-1* heterozygotes in W22 background and A188, B73, Mo17, Oh51a, or WT plant from the *why1-1* segregating line. Arrow points to the defective embryo in *emb* kernel.

Intriguingly, the *Why1* gene was isolated from an *albino* seedling mutant [33]. The suspected severe allele of *why1-1* is capable to develop viable seeds, indicating normal embryogenesis. The different phenotypes in *why1-1* and *emb16* raised the possibility that the *emb16* phenotype may be caused by another mutation that is tightly linked to *why1*. To test this possibility, we obtained the previous *why1-1* and *why1-2* alleles from Dr. Alice Barkan [33]. The strong allele *why1-1* carrying a *MuDR* insertion 35 bps downstream of the *Why1* translation start codon conditions ivory seedlings; the weak allele *why1*-*2* carrying a *Mu1* or *Mu2* insertion in the 5′-UTR of the *Why1* gene conditions pale green seedlings. We crossed *emb16* heterozygotes with the *why1-1* and *why1-2* heterozygotes, respectively. The F1 of the *emb16*/+×*why1-1*/+ crosses segregated mostly ivory seedlings, but also *emb* mutants (Figure 5). The sum of (*emb*+*albino*) accounted for ∼25% of the total kernels, indicating a single recessive mutation. The crosses of *emb16*/+×*why1-2*/+ segregated only pale green seedlings (Figure 5A). The ivory and pale green seedlings in all the crosses were genotyped and confirmed to be compound heterozygotes, *i.e. emb16:why1-1* or *emb16:why1-2*. Because these crosses produced *albino* seedlings and/or *emb* kernels, this result confirms that the mutation in the *Why1* gene is the cause of the *emb16* phenotype. We therefore named the *emb16* mutant *why1-3*.

### The *why1-3* Phenotypic Expression is Dependent on Genetic Backgrounds

**Figure 6:**
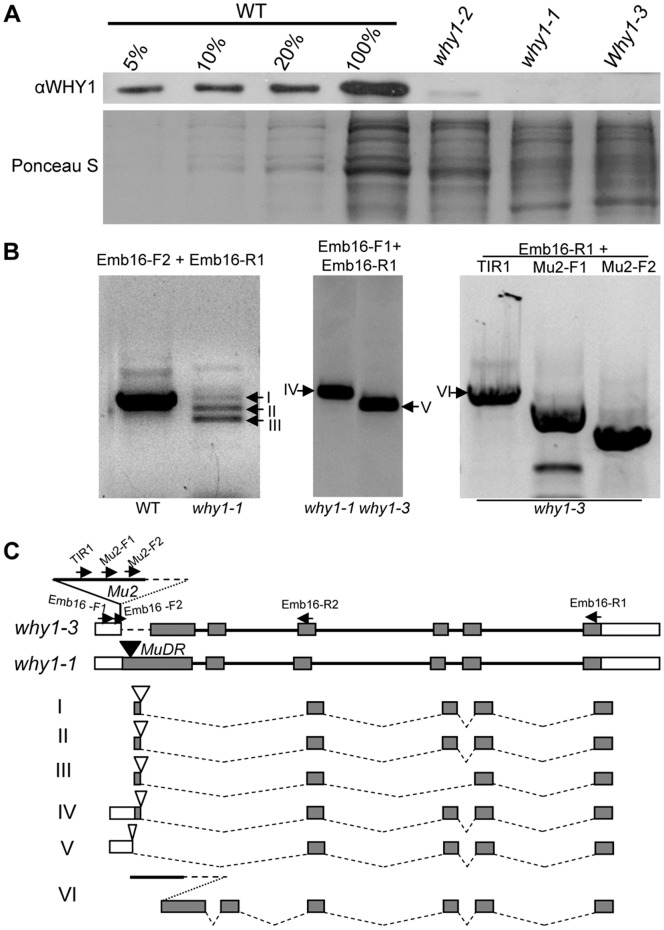
Expression studies of *Why1* gene in seedlings homozygous for *why1-1*, *why1-2*, or *why1-3*. (A) Immunoblot analysis on WHY1 protein in the mutant leaf tissue. Total leaf extract of 10 µg protein, or dilutions as indicated were analyzed. The same blot was stained with Ponceau S. (B) RT-PCR analysis on WT seedling, and the *albino* seedlings of *why1-1* and *why1-3* using primers as indicated. TIR1, Mu2-F1, and Mu2-F2 primers are nested primers in *Mu2*. Arrows point to fragments recovered and sequenced. (C) The alternative spliced forms of the *why1* gene in the *albino* seedlings homozygous for *why1-1* or *why1-3*. The primer sites are indicated by arrows. Empty triangles are the spliced *Mu* element.

The different phenotypic expression of *why1* mutation invokes two explanations, 1) *why1-3* is a null allele, whereas *why1-1* is leaky; 2) the genetic backgrounds determine the phenotypic expression of *why1* mutation. The first possibility assumes that a low level of WHY1 is sufficient for embryogenesis. To examine the WHY1 protein expression levels, we performed Western blot analysis on seedling leaves of homozygous for *why1-1*, *why1-2* or *why1-3*. The WHY1 antibody (generously provided by Dr. Alice Barkan) detected a single band with expected size of WHY1 (∼26 kD) in both WT and *why1-2*, but not in *why1-1* and *why1-3*, suggesting that *why1-2* is a leaky allele and both *why1-1* and *why1-3* are likely null alleles (Figure 6A). The leaky nature in *why1-2* is consistent with the *Mu* insertion 38 bps upstream of the translation start codon which may allow leaky expression [33]. To analyze the *why1* transcripts in these alleles, RT-PCR was performed. In *why1-1* ivory leaves, four major fragments were detected and sequenced (Figure 6B). The results indicate that these transcripts are all incorrectly spliced, removing a major part of the *MuDR* element and most of the first exon and the entire second exon of *Why1*. None of these transcripts could predict a likely functional WHY1 protein (Figure 6C). In the *why1-3* allele, two transcripts were detected (Figure 6, B and C). One could not predict a functional protein. The other contained the *Mu2* element which could predict a fusion protein with the N-terminus encoded by the *Mu2* element. However, due to the deletion in the first exon of *Why1* that removed the transit peptide, this fusion protein is unlikely to target itself to the chloroplast. Supporting this conclusion, neither the ChloroP [48] nor the Predotar [49] algorithms predict a transit peptide in this protein. This analysis suggests that *why1-1* and *why1-3* are likely null alleles. The null nature of *why1-3* mutation is consistent with the deletion in the first exon that removes the transit peptide. However, the *why1-1* allele was reported previously to have a low level expression [33]. There were multiple fragments in the RT-PCR analysis of *why1-1* ivory leaves (Figure 6B). For that reason, we cannot completely rule out the possibility that the expression level difference contributed to the phenotypic difference.

We then tested whether genetic backgrounds could explain the different phenotypic expression of these alleles. The *why1-3* heterozygotes in the W22 background were crossed with inbreds A188, B73, Mo17, Oh51a and the WT *Why1-1* (Figure 5B). The F2 progenies of these crosses segregated *emb* kernels and *albino* seedlings. And reciprocal crosses produced the same result. The ratio of the *emb* and *albino* mutants together is ∼25% of all seeds, consistent with one recessive mutation (Table 1). The *albino* seedlings were confirmed to be homozygous *why1-3* by PCR genotyping. This result indicates that the *why1-3* mutation could condition *albino* seedling (normal embryogenesis) or embryo lethal phenotype, which is dependent on the genetic backgrounds.

**Table 1:** Ratio of *emb* kernels plus *albino* seedlings from the F2 progenies of the crosses between *emb* heterozygotes (*why1-3*/+, *emb12-1*/+, and *emb14-1*/+) in W22 background and maize inbred lines (A188, B73 or Mo17), and goodness-of-fit test for a monogenic inheritance.

Crosses in F1 (♀×♂)	Total ratio of *emb* and *albino*	Total no. of *emb* and *albino*	Total no. of WT	Expected ratio	*x2*	*P-value*
*why1-3*/+×A188	26.3%	97	272	1∶3	0.26	0.50–0.75
A188×*why1-3*/+	25.0%	37	111	1∶3	0	1
*why1-3*/+×B73	29.6%	165	393	1∶3	5.97	0.01–0.05
*why1-3*/+×Mo17	24.7%	390	1190	1∶3	0.07	0.75–0.90
Mo17×*why1-3*/+	26.4%	288	803	1∶3	1.06	0.25–0.50
*emb12-1*/+×B73	26.6%	143	395	1∶3	0.63	0.25–0.50
*emb12-1*/+×Mo17	23.9%	67	213	1∶3	0.12	0.50–0.75
*emb14-1*/+×B73	23.7%	89	286	1∶3	0.26	0.50–0.75
B73×*emb14-1*/+	27.2%	205	550	1∶3	1.75	0.10–0.25
*emb14-1*/+×Mo17	25.2%	148	440	1∶3	0	>0.95
Mo17×*emb14-1*/+	23.8%	31	99	1∶3	0.04	0.75–0.90

We also crossed *why1-1* allele from its original background to W22 background by continued backcrossing. No *emb* kernels were obtained in the selfed progenies of the first, second and third backcross generations. One possible explanation is that *why1-1* is leaky as indicated previously [33], such that a small amount of WHY1 protein is sufficient for embryogenesis regardless of the genetic background. For that reason, the genetic background dependence of embryogenesis may be masked in the *why1-1* allele.

### Plastid Translation Mutants Show Genetic Background Dependence for Either *emb* Kernel or *albino* Seedling Phenotypes

One molecular consequence of the *why1-1* mutation is deficiency in plastid ribosome formation, thus the mutant abolishes protein translation of the plastid encoded genes [33]. We speculate that the genetic background dependence for embryo lethality may not be unique to *why1*, but a shared feature for other plastid translation mutants as well. To test this notion, we crossed two embryo defective mutations, the *emb12-1* and *emb14-1* heterozygotes with B73 and Mo17 inbreds. The *emb12-1* and *emb14-1* alleles were isolated from the UniformMu population, hence in a nearly isogenic W22 genetic background (Figure 5A). *Emb12* encodes the plastid translation initiation factor 3 [12]. *Emb14* encodes an YqeH homolog that shows significant similarity to nitric oxide associated 1 in rice and *Arabidopsis* (Li C. and Tan, B.C., unpublished data). It was believed to function in the ribosome assembly in plastids [50]. Loss of function mutants in *Emb12* or *Emb14* showed a similar embryo arrest as the *why1-3*. Also similar with *why1-3*, both *emb* kernels and *albino* seedlings were obtained in the F2 progenies of all the crosses (Figure 5B), and the ratio of *emb* plus *albino* mutants was ∼25% (Table 1). Reciprocal crosses showed the same result. PCR genotyping confirmed the *albino* seedlings were homozygous for *emb12-1* or *emb14-1* allele. These results indicate that the *emb12* and *emb14* mutations also condition *albino* phenotype in B73 and Mo17 backgrounds.

### Chloroplast Biogenesis is Arrested in the *why1* Mutant

**Figure 7:**
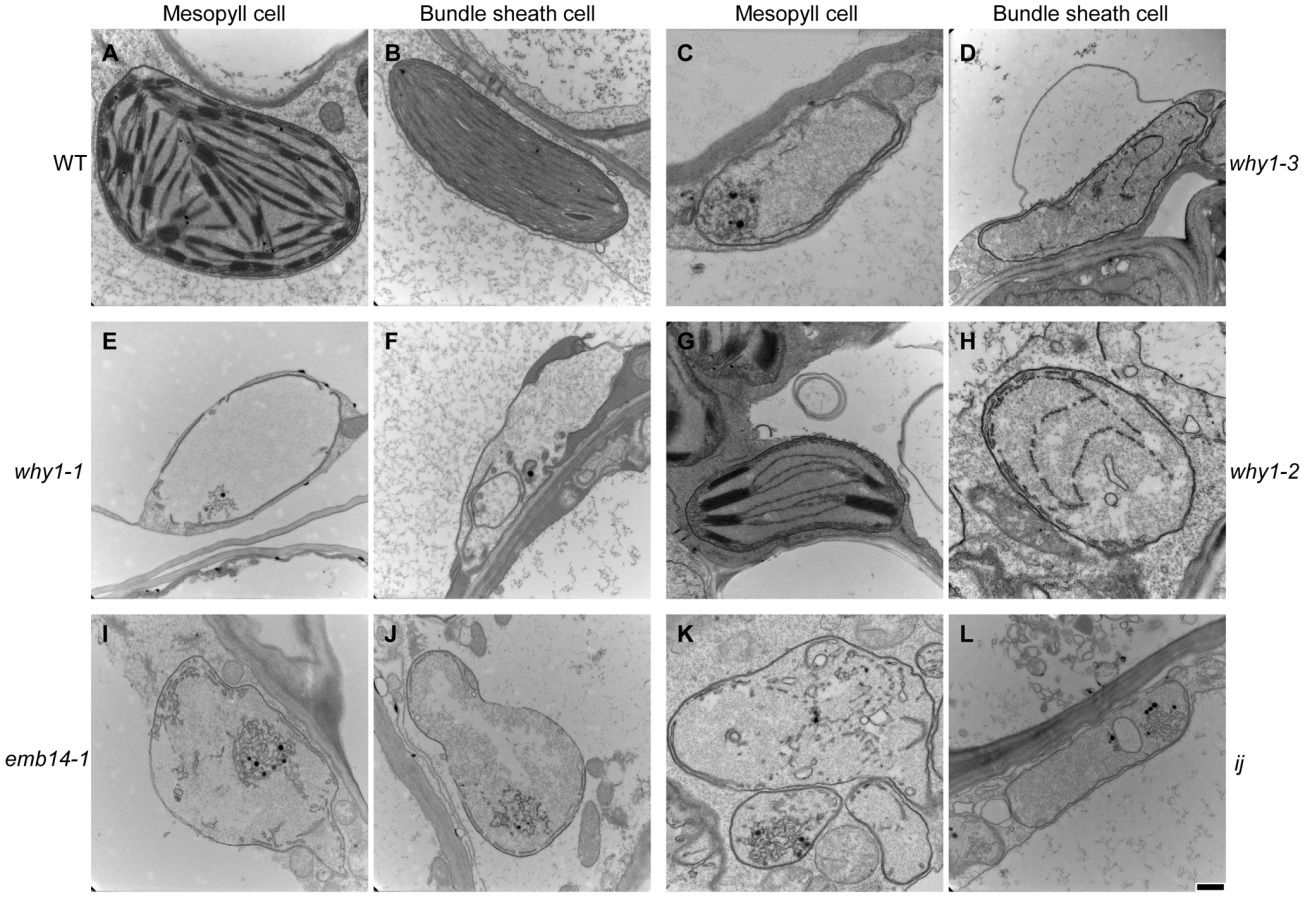
Chloroplast in seedlings homozygous for *why1-1*, *why1-2*, *why1-3*, *emb14-1*, or *ij*. Leaf sector 1 cm below the tip of the second leaf from two-leaf stage seedlings were fixed and sectioned for TEM. Scale bars = 0.5 µm.

The function of WHY1 in maize has not been well understood although several studies have been reported [31], [33]. The chloroplast localization of WHY1 promoted us to examine the impact on chloroplast biogenesis in the absence of WHY1 protein. As shown in Figure 7, when compared with the WT, the thylakoid biogenesis was found impaired in all three alleles, and the severity of the impact was consistent with severity of mutation, *i.e.* more severely arrested in *why1-1* and *why1-3* alleles than in the *why1-2* leaky allele. In *why1-2*, most of the thylakoids were stacked to form grana in mesophyll cells, but no grana formation was observed in *why1-1* and *why1-3*, which was due to the low amount of thylakoid. In contrast to the embryo cells of *why1-3* *emb* kernels, the number of mitochondria in the leaf cells was comparable between the mutants and the WT. Similar to *why1-1* and *why1-3*, the biogenesis of thylakoid was also severely reduced in the *albino* seedlings of *emb14-1* and *ij* (Figure 7, I-L; [51]). Since IJ protein is also required for plastid translation [33], these results suggest that plastid translation is required for biogenesis of thylakoid membranes, and WHY1 may negatively regulate the stacking of thylakoids to form granas.

### Dual-localization of WHY1

**Figure 8:**
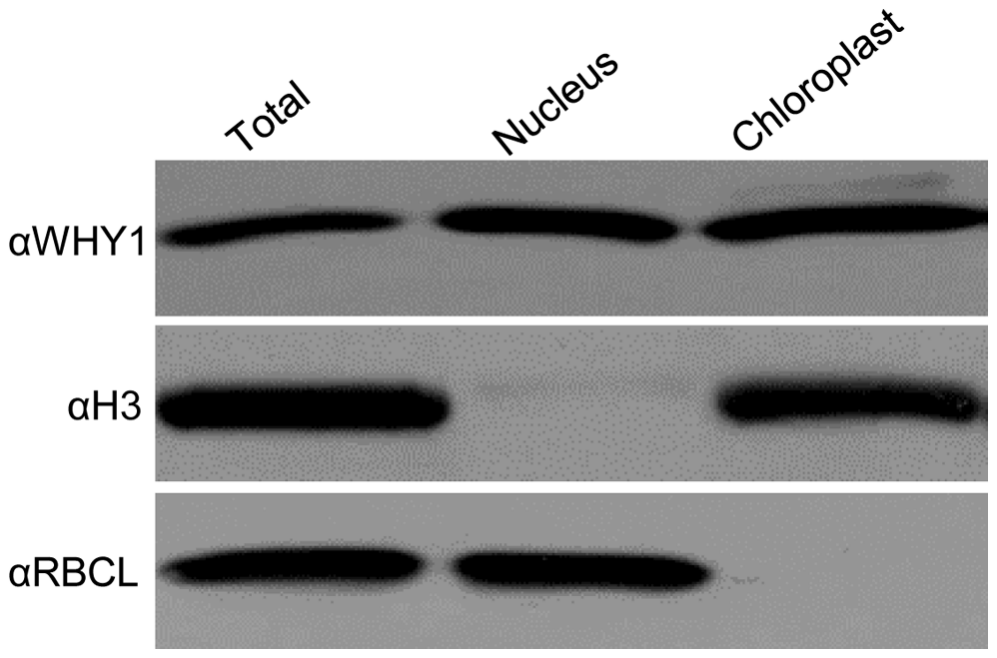
Intracellular localization of WHY1 protein in maize. Immunoblots of extracts from leaf and subcellular fractions: nucleus and chloroplast. The same blot was probed to detect a marker for nucleus (H3) and chloroplast (RBCL).

The maize WHY1 protein was previously localized in the chloroplast [33]. However, orthologous WHY1 proteins in *Arabidopsis* and barley were shown to be dual-localized in the chloroplast and the nucleus [26], [27]. To test the nuclear localization of maize WHY1, immunodetection was performed. The nuclei and chloroplasts were purified from W22 seedling leaves and the proteins were extracted. The protein blot was hybridized by WHY1 antibody. Cross-contamination was monitored by the chloroplast marker RUBISCO large subunit (RBCL) and the nuclear marker histone 3 (H3). As shown in Figure 8, the WHY1 antibody recognized a single 27 kD protein in both the nuclear and chloroplast fractions. The size of WHY1 in the chloroplast is close to that in the nucleus, suggesting that WHY1 may have either a short or no transit peptide. No cross contamination was detected between the two fractions. This result indicates that maize WHY1, similar to its orthologs in *Arabidopsis* and barley, is dual-localized in the plastid and the nucleus.

## Discussion

Through the molecular characterization, we revealed that the arrest of embryogenesis in the maize *emb16* mutant is caused by a mutation in *Why1* gene, thus demonstrating that embryogenesis requires the function of *Why1*. WHY1 has been implicated in modulating the homeostasis of telomere length and activating or repressing transcription in the nucleus [25], [28]–[30], and genome stability and ribosome formation in the plastid [31], [33]. However, its molecular function is still unclear. Genetic analyses revealed that the requirement of WHY1 function for embryogenesis can be suppressed in maize A188, B73, Mo17 and Oh51a inbreds, giving rise to an *albino* seedling phenotype. And similar suppression was found in *emb12* and *emb14* mutants which were impaired in plastid translation process. Given that *why1* mutants are deficient in plastid ribosomes [33], these results indicate that the requirement of plastid translation for embryogenesis can be suppressed by a likely common mechanism.

This genetic suppression of embryo lethality offers an explanation to the relationship between plastid translation and embryogenesis. In *Arabidopsis*, mutations impairing plastid translation process cause embryo lethality [6]–[8]. However, in maize, mutations abolishing plastid ribosome assembly and translation gave rise to three phenotypes, *i.e.* lethal embryo, *albino* seedling and stripped leaves [10]–[12], [16]–[19], [33], [51]. The first class of mutants demonstrates an essential function of plastid translation to embryogenesis. Whereas the last two classes of mutants indicate that embryogenesis does not require the expression of the entire plastid genome because all these mutants have normal embryogenesis and produce viable seeds. Given our results on *why1*, *emb12* and *emb14*, it is likely that the *albino* seedling and striping leaf phenotypes are conditioned in specific genetic backgrounds where embryo lethality is suppressed. Previous studies of the plastid ribosome deficient leaf striping mutant *ij* have revealed evidence of genetic suppressors [33], [51]. In K55 and Ky21 genetic backgrounds, *ij* conditions embryo lethality, but seedlings with stripe leaves in Mo17 and Oh51a backgrounds. Similar suppression was observed in *emb8522*, which conditions embryo lethality in the original genetic background but in A188 and B73 backgrounds conditions *albino* seedlings [34]. *Emb8522* encodes a plastid PPR protein with possible functions in plastid gene expression. Together, these results suggest that the genetic background difference in maize is a key factor that contributes to the unequal requirement of plastid translation for embryogenesis in maize and *Arabidopsis*.

This genetic background determination of the requirement of plastid translation for embryogenesis implies a genetic mechanism mediating this process. One puzzle in embryogenesis in flowering plants is to understand what factors constitute the requirement of plastid translation for embryogenesis. One hypothesis assumes that specific products encoded by the plastid genome are required for embryogenesis [6], [34], and another hypothesis assumes that a defect in plastid protein translation triggers the release of a retrograde signal to shut down the embryogenesis process [12]. These two hypotheses are not mutually exclusive. In *Arabidopsis*, the *accD*, *clpP*, *ycf1* and *ycf2* genes in the plastid genome have been considered [6], [52], [53]. But in maize, the *accD*, *ycf1* and *ycf2* genes no longer exist in the plastid genome, and yet defective plastid translation mutations still cause embryo lethality [10]–[12], [54]. This evidence argues that these gene products are not the factors required by embryogenesis, at least in maize. The requirement of *clpP* was also challenged because unedited *clpP* did not cause embryo lethality [55]. Sosso et al. proposed that the plastid *trnE* gene encoding tRNA-Glu may be the plastid factor [34]. Besides protein translation, tRNA-Glu is the substrate for haem synthesis and haem is an essential prosthetic group of many important proteins in plastids and mitochondria. However, haem biosynthetic mutants did not condition embryo lethality in maize and *Arabidopsis* [56], [57].

Our results favor the retrograde signaling hypothesis. The suppression of embryo lethality in *why1*, *emb12* and *emb14* in certain genetic backgrounds suggests the presence of suppressor(s) that can suppress the requirement of plastid translation for embryogenesis. Maize is known for its wide diversity [58]–[60]. Inbred B73 and Mo17 are different in copy numbers in several hundred sequences and presence/absence variations in several thousand sequences [58], and 4–18% genes with differential expression patterns [59]. This diversity renders the possibility that a functional homolog with overlapping expression of *Why1* in certain inbreds that confers the suppressor function. We reason this possibility is unlikely because: 1) we did not find another copy of *Why1* in the sequenced B73 genome; 2) *Why1* (chr6), *Emb12* (chr5) and *Emb14* (chr4) locate on different chromosomes. Thus, all three genes should have at least one paralog or one homolog in the B73 genome, for which we did not find in the sequenced genome. Genomic DNA hybridization confirmed only one copy of *Emb12* in the B73 genome [12]. Overwhelming evidence supports the existence of a retrograde signaling pathway coordinating the plastid and the nuclear gene expression [61]. This signal may be associated with the plastid translation machinery to monitor its integrity. A defect in plastid translation machinery triggers its release and a shut-down of the cell activity, thus causing embryo lethality. Along with this reasoning, this pathway may be fully functional in the W22 genetic background but dysfunctional in A188, B73, Mo17 and Oh51a genetic backgrounds as a result of natural mutations. This would provide a plausible explanation for the suppression of embryo lethality in some maize genetic backgrounds, but not in others.

WHY1 orthologous proteins in barley and *Arabidopsis* are localized in the plastid and the nucleus, with majority of the proteins targeted to plastids [26], [27]. The maize WHY1 showed a similar dual localization (Figure 8). The *why1-3* allele in the W22 genetic background conditions specific arrest in embryogenesis without major impact on endosperm development. This phenotype is found in *lem1*, *emb8516* and *emb12* mutant [10]–[12], all of which are implicated in plastid function. This consistency suggests that the embryo defective phenotype of *why1-3* is likely due to its plastid function because the *why1* mutation abolishes the ribosome formation [33]. Comparing with the arrested embryogenesis, the endosperm development appears to be less dependent on the plastid gene expression in these mutants regardless of the genetic backgrounds. This difference may be related to the different fates of proplastids in the endosperm and the embryo, which was discussed previously [12]. Considering the results of this study, it is also possible that the retrograde signaling pathway in the embryo is not present in the endosperm. As such, the endosperm does not produce the signal even though the plastid translation is defective. In any case, identifying the suppressors of embryo lethality in plastid translation mutants is the key to understanding the plastid function in embryogenesis and plant development.

## Supporting Information

Figure S1 **The inward development of aleurone cells in the kernel germinal face of** ***emb16*** **mutant.** The WT and *emb16* mutant kernels from a segregating ear were sectioned from 14 to 27 DAP. Arrows point to abnormally dividing aleurone cells in *emb16* mutant. al: aleurone cells; s: seed coat; sc: scutellum; se: starchy endosperm; ep: embryo proper. Scale bars = 0.1 mm.
(PPT)

Figure S2 **The development of proplastid and mitochondrion in** ***emb16*** **mutant.** The WT and *emb16* mutant kernels from segregating ears were sectioned from 6 to 14 DAP. At 6 DAP, *emb16* embryo is distinguished from WT by the size and structure of embryo proper using stereomicroscopy and confirmed by the endosperm genotyping. The ultrastructural observation of embryo cells for *emb16* mutant is from the embryo proper cells and for WT is from embryo proper cells (6 DAP) or shoot meristem cells (7 and 8 DAP). Embryo proper cells are different from suspensor cells, which contain more starch granules and vacuoles. Similar cell contents were observed in cells of shoot meristem, leaf primordia, and coleoptile in the WT embryo. Empty arrow heads point to mitochondria, and filled arrow heads point to proplastids. Scale bars = 0.5 µm.
(PPT)

Table S1 **Primers used in this paper.**
(PPT)
