# Supplementary figures and images for: The Requirement of WHIRLY1 for Embryogenesis Is Dependent on Genetic Background in Maize
Source: PLoS One. 2013 Jun 28;8(6):e67369. doi: 10.1371/journal.pone.0067369 (PMC3696099; doi:10.1371/journal.pone.0067369)

## Slide 1
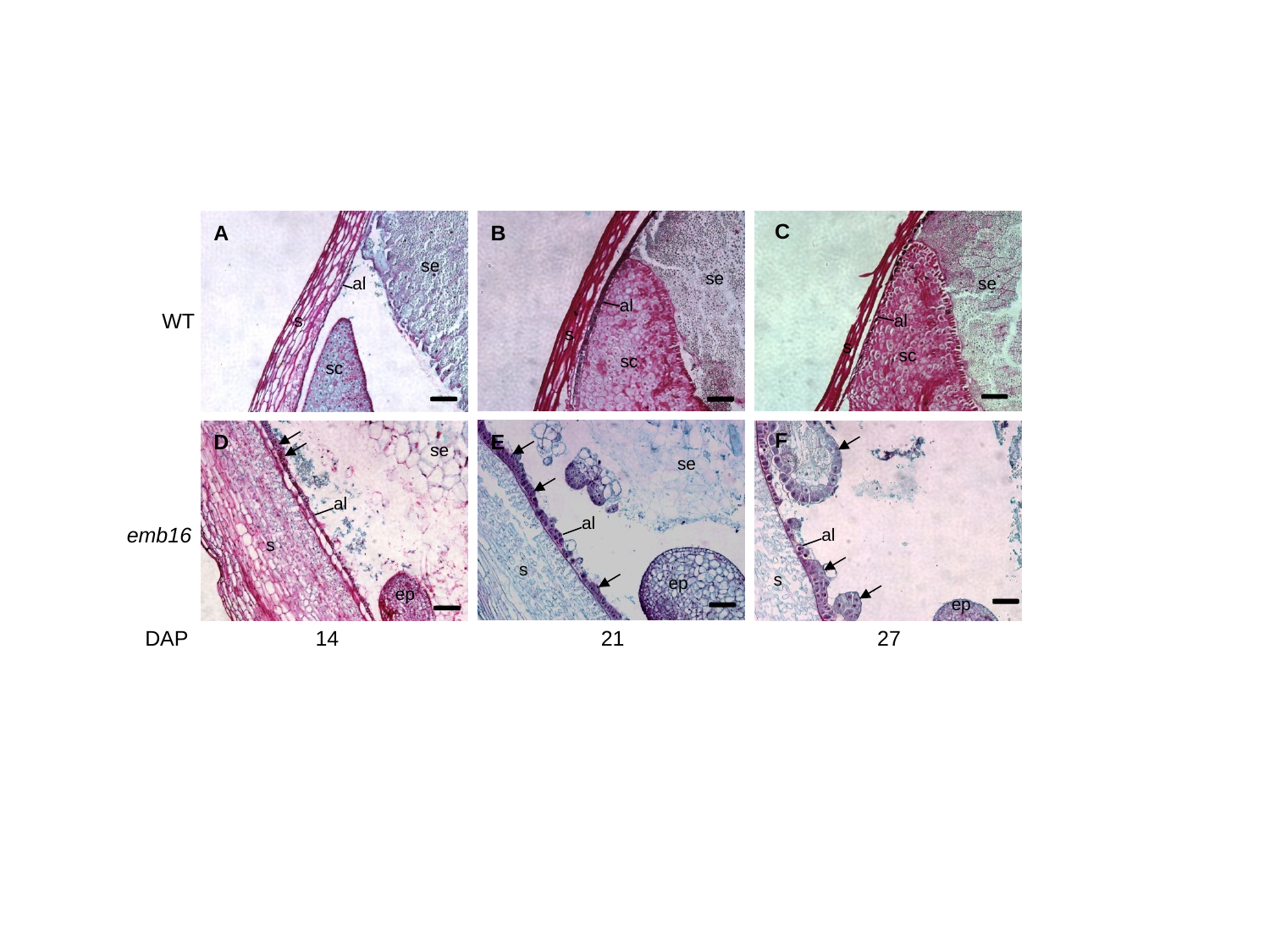

C
A
B
se
se
al
se
al
WT
al
s
s
s
sc
sc
sc
F
D
E
se
se
al
al
emb16
al
s
s
s
ep
ep
ep
DAP
14
21
27

Supplement: Figure S1 — The inward development of aleurone cells in the kernel germinal face of emb16 mutant. The WT and emb16 mutant kernels from a segregating ear were sectioned from 14 to 27 DAP. Arrows point to abnormally dividing aleurone cells in emb16 mutant. al: aleurone cells; s: seed coat; sc: scutellum; se: starchy endosperm; ep: embryo proper. Scale bars = 0.1 mm. (PPT) [file pone.0067369.s001.ppt]

## Slide 1
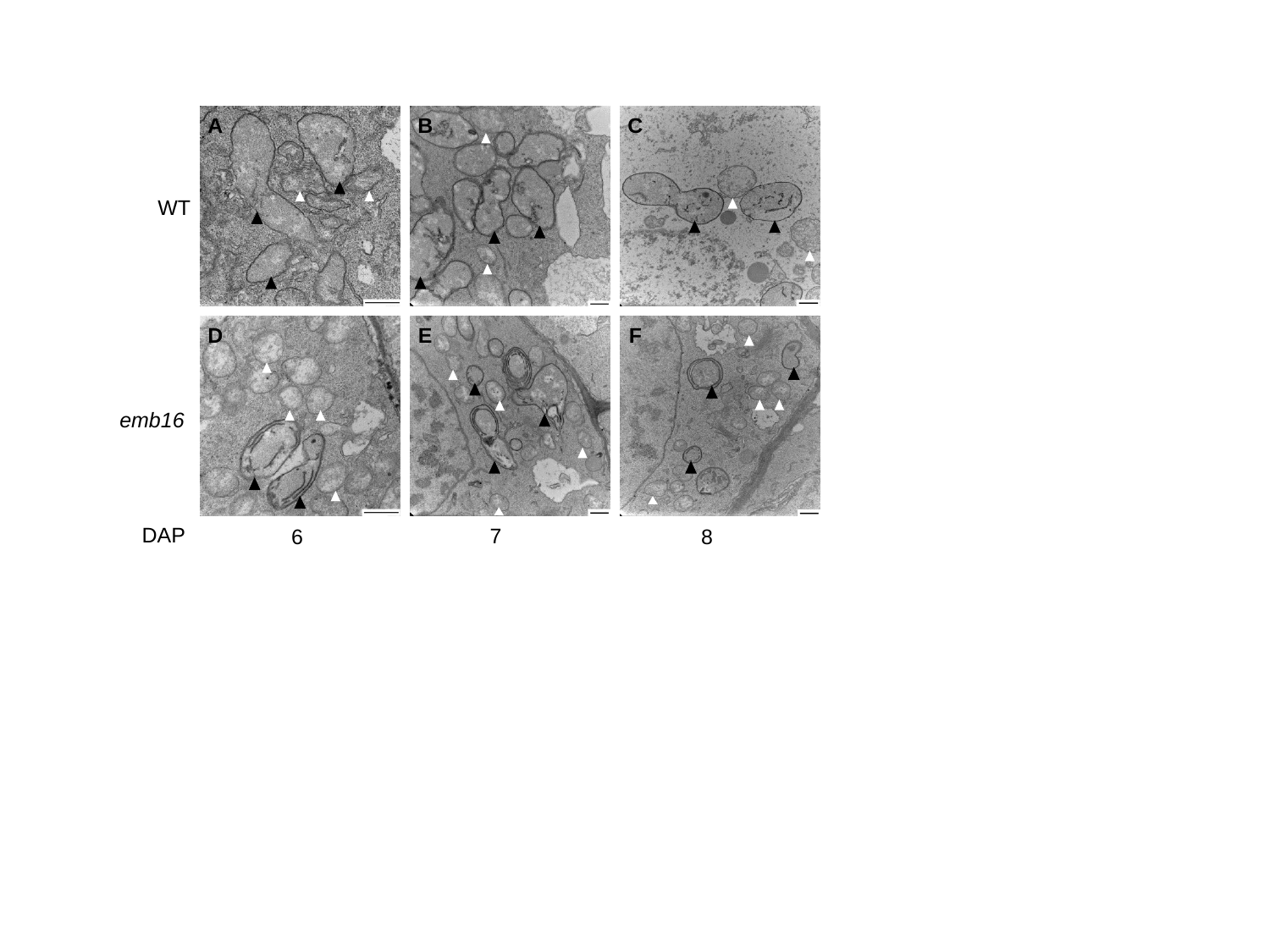

A
B
C
WT
D
E
F
emb16
DAP
7
6
8

Supplement: Figure S2 — The development of proplastid and mitochondrion in emb16 mutant. The WT and emb16 mutant kernels from segregating ears were sectioned from 6 to 14 DAP. At 6 DAP, emb16 embryo is distinguished from WT by the size and structure of embryo proper using stereomicroscopy and confirmed by the endosperm genotyping. The ultrastructural observation of embryo cells for emb16 mutant is from the embryo proper cells and for WT is from embryo proper cells (6 DAP) or shoot meristem cells (7 and 8 DAP). Embryo proper cells are different from suspensor cells, which contain more starch granules and vacuoles. Similar cell contents were observed in cells of shoot meristem, leaf primordia, and coleoptile in the WT embryo. Empty arrow heads point to mitochondria, and filled arrow heads point to proplastids. Scale bars = 0.5 µm. (PPT) [file pone.0067369.s002.ppt]
