# Supplementary material for: The Requirement of WHIRLY1 for Embryogenesis Is Dependent on Genetic Background in Maize
Source: PLoS One. 2013 Jun 28;8(6):e67369. doi: 10.1371/journal.pone.0067369 (PMC3696099; doi:10.1371/journal.pone.0067369)
Supplement: Table S1 — Primers used in this paper. (PPT) [file pone.0067369.s003.ppt]

## Slide 1
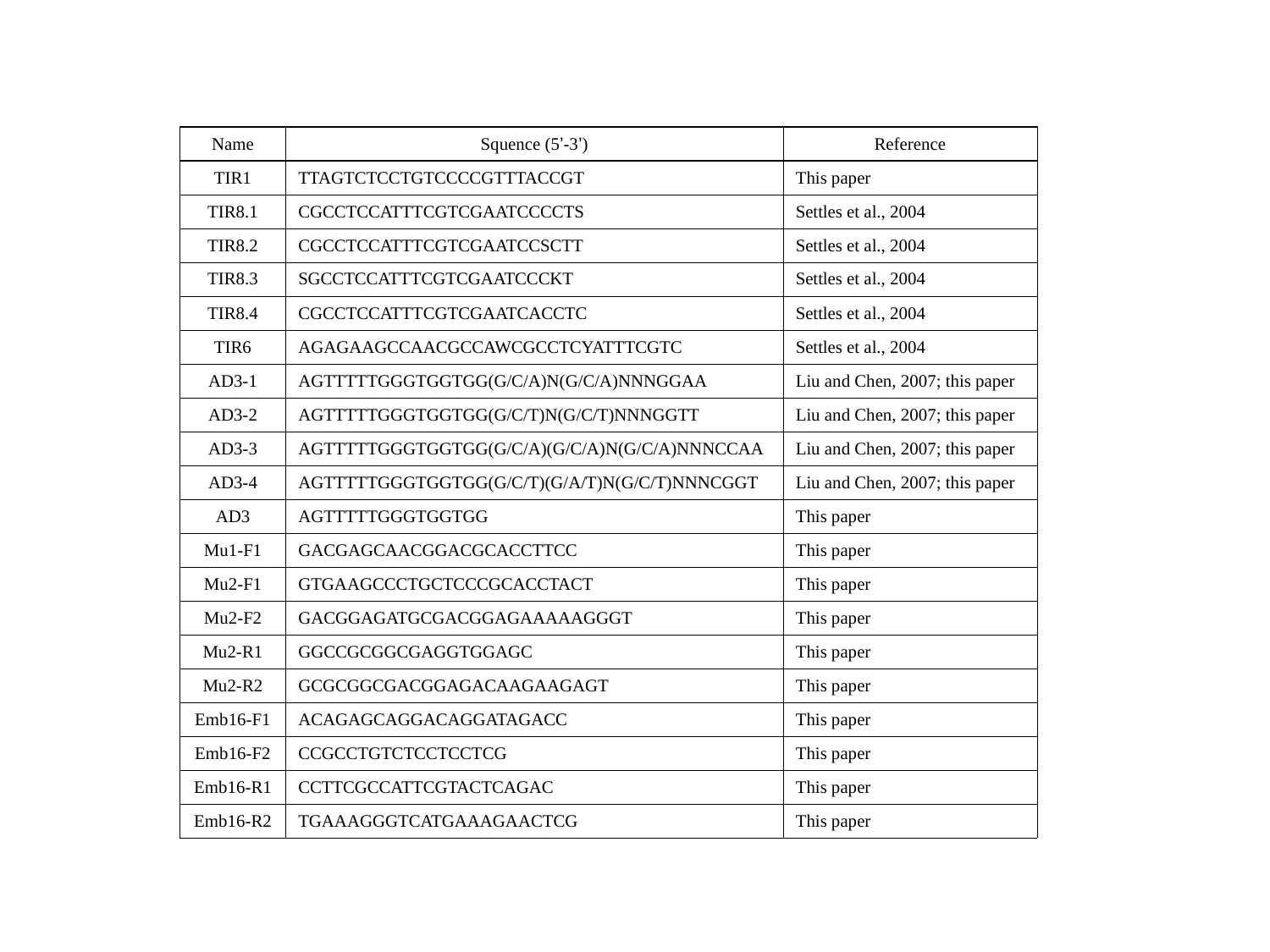

| Name | Squence (5’-3’) | Reference |
| --- | --- | --- |
| TIR1 | TTAGTCTCCTGTCCCCGTTTACCGT | This paper |
| TIR8.1 | CGCCTCCATTTCGTCGAATCCCCTS | Settles et al., 2004 |
| TIR8.2 | CGCCTCCATTTCGTCGAATCCSCTT | Settles et al., 2004 |
| TIR8.3 | SGCCTCCATTTCGTCGAATCCCKT | Settles et al., 2004 |
| TIR8.4 | CGCCTCCATTTCGTCGAATCACCTC | Settles et al., 2004 |
| TIR6 | AGAGAAGCCAACGCCAWCGCCTCYATTTCGTC | Settles et al., 2004 |
| AD3-1 | AGTTTTTGGGTGGTGG(G/C/A)N(G/C/A)NNNGGAA | Liu and Chen, 2007; this paper |
| AD3-2 | AGTTTTTGGGTGGTGG(G/C/T)N(G/C/T)NNNGGTT | Liu and Chen, 2007; this paper |
| AD3-3 | AGTTTTTGGGTGGTGG(G/C/A)(G/C/A)N(G/C/A)NNNCCAA | Liu and Chen, 2007; this paper |
| AD3-4 | AGTTTTTGGGTGGTGG(G/C/T)(G/A/T)N(G/C/T)NNNCGGT | Liu and Chen, 2007; this paper |
| AD3 | AGTTTTTGGGTGGTGG | This paper |
| Mu1-F1 | GACGAGCAACGGACGCACCTTCC | This paper |
| Mu2-F1 | GTGAAGCCCTGCTCCCGCACCTACT | This paper |
| Mu2-F2 | GACGGAGATGCGACGGAGAAAAAGGGT | This paper |
| Mu2-R1 | GGCCGCGGCGAGGTGGAGC | This paper |
| Mu2-R2 | GCGCGGCGACGGAGACAAGAAGAGT | This paper |
| Emb16-F1 | ACAGAGCAGGACAGGATAGACC | This paper |
| Emb16-F2 | CCGCCTGTCTCCTCCTCG | This paper |
| Emb16-R1 | CCTTCGCCATTCGTACTCAGAC | This paper |
| Emb16-R2 | TGAAAGGGTCATGAAAGAACTCG | This paper |
